# Physical Activity and Sedentary Time Among U.S. Adolescents Before and During COVID-19: Findings From a Large Cohort Study

**DOI:** 10.1016/j.focus.2024.100253

**Published:** 2024-06-17

**Authors:** Ethan T. Hunt, Keith Brazendale, Augusto C.F. De Moraes, Raja Malkani, Natalia I. Heredia, Christopher D. Pfledderer, Denver M. Brown, Deanna M. Hoelscher, Michael W. Beets, Robert G. Weaver

**Affiliations:** 1 Michael and Susan Dell Center for Healthy Living, University of Texas Health Science Center at Houston (UTHealth) School of Public Health, Austin, Texas; 2 Department of Health Promotion and Behavioral Science, University of Texas Health Science Center at Houston (UTHealth) School of Public Health, Austin, Texas; 3 Department of Health Sciences, UCF College of Health Professions and Sciences, University of Central Florida, Orlando, Florida; 4 Department of Epidemiology, Human Genetics and Environmental Science, University of Texas Health Science Center at Houston (UTHealth) School of Public Health, Austin, Texas; 5 Department of Psychology, The University of Texas at San Antonio, San Antonio, Texas; 6 Department of Exercise Science, Arnold School of Public Health, University of South Carolina, Columbia, South Carolina

**Keywords:** COVID-19, physical activity, adolescents, summer

## Abstract

**Introduction:**

Evidence suggests that adolescents engage in less physical activity during the summer break. Less is known regarding physical activity during the summer months of the COVID-19 pandemic.

**Methods:**

Utilizing data from the Adolescent Brain Cognitive Development study, the authors examined daily activity measured by Fitbit Charge 2 devices before and after the onset of the COVID-19 pandemic during school and summer months. Linear models estimated activity during pre–COVID-19 school, pre–COVID-19 summer, COVID-19 school, and COVID-19 summer.

**Results:**

Participants (N=7,179, aged 11.96 years, 51% female, 51% White) accumulated 8,671.0 (95% CI=8,544.7; 8,797.3) steps, 32.5 (95% CI=30.8, 32.3) minutes of moderate-to-vigorous physical activity, and 507.2 (95% CI=504.2, 510.2) minutes of sedentary time. During COVID-19 school, adolescents accumulated fewer daily steps and minutes of moderate-to-vigorous physical activity (−1,782.3 steps [95% CI= −2,052.7; −1,511.8] and −6.2 minutes [95% CI= −8.4, −4.0], respectively). Adolescents accumulated more minutes of daily sedentary time (29.6 minutes [95% CI=18.9, 40.3]) during COVID-19 school months than during the pre–COVID-19 school months. During pre–COVID-19 summer months, adolescents accumulated 1,255.1 (95% CI=745.3; 1,765.0) more daily steps than during COVID-19 months. Boys accumulated more daily steps and moderate-to-vigorous physical activity (2,011.5 steps [95% CI=1,271.9; 2,751.0] and 7.9 minutes [95% CI=1.4, 14.4], respectively) during the summer before COVID-19 than in summer during COVID-19. Both girls and boys accumulated more minutes of sedentary time during COVID-19 school months (47.4 [95% CI=27.5, 67.3] and 51.2 [95% CI=22.8, 79.7], respectively) than during COVID-19 summer months.

**Conclusions:**

Societal restrictions during COVID-19 negatively impacted activity levels in the U.S., particularly during the summer months during COVID-19.

## Highlights

• The COVID-19 pandemic negatively impacted physical activity among U.S. adolescents.
• During COVID-19 summer, adolescents' steps dropped compared with those during pre–COVID-19 school.
• Both boys and girls had less moderate-to-vigorous physical activity during COVID-19 school than during pre–COVID-19 school.

## INTRODUCTION

Physical activity (PA), including steps and sedentary time, plays a critical role in the physical and mental health of youth.^1^^,^^2^ Considerable evidence has also shown that on days when children and adolescents attend a structured setting, such as school during the school year and extracurricular programs or day camps during the summer, their daily PA levels are higher than on days when they do not attend, such as weekend days.^3^, ^4^, ^5^, ^6^, ^7^, ^8^, ^9^, ^10^, ^11^, ^12^, ^13^ A recent systematic review concluded that adolescent health behaviors are less healthy on days with less structure.^6^

Throughout the coronavirus disease 2019 (COVID-19) period, state and national lockdowns were implemented in the U.S. to mitigate and prevent the spread of the disease. These lockdowns produced societal restrictions, such as the closure of schools, which significantly disrupted the day-to-day environments.^14^ Evidence from this period has shown these restrictions and closures dramatically altered adolescent behavior, with studies reporting increases in screen time, reductions in PA, and more irregular sleep patterns.^15^, ^16^, ^17^, ^18^, ^19^, ^20^, ^21^ Such unfavorable changes in health behaviors have raised concerns about the potential long-term impact on adolescents' overall health and well-being. Furthermore, additional evidence examined during the pandemic revealed sex differences among adolescents on numerous health outcomes, including PA.^16^^,^^22^ Recent research has not fully explored the effects of the COVID-19 lockdown on PA patterns among U.S. adolescents. There is a need for studies that compare levels of objectively measured activity before and during the COVID-19 pandemic, specifically including the summer break period in the U.S. This study aims to examine the patterns of objectively measured steps, moderate-to-vigorous PA (MVPA), and sedentary time among a sample of U.S. adolescents over 2 years before and during COVID-19. The authors further aim to explore whether a double jeopardy exists when examining summer months during COVID-19 compared with that before the COVID-19 pandemic. The authors hypothesize that adolescent PA will be lower (less MVPA, fewer steps, increased sedentary time) after the onset of the COVID-19 pandemic and the lowest during less-structured periods in comparison with both school year months and summer months before the COVID-19 pandemic.

## METHODS

### Study Sample

**Figure 1:**
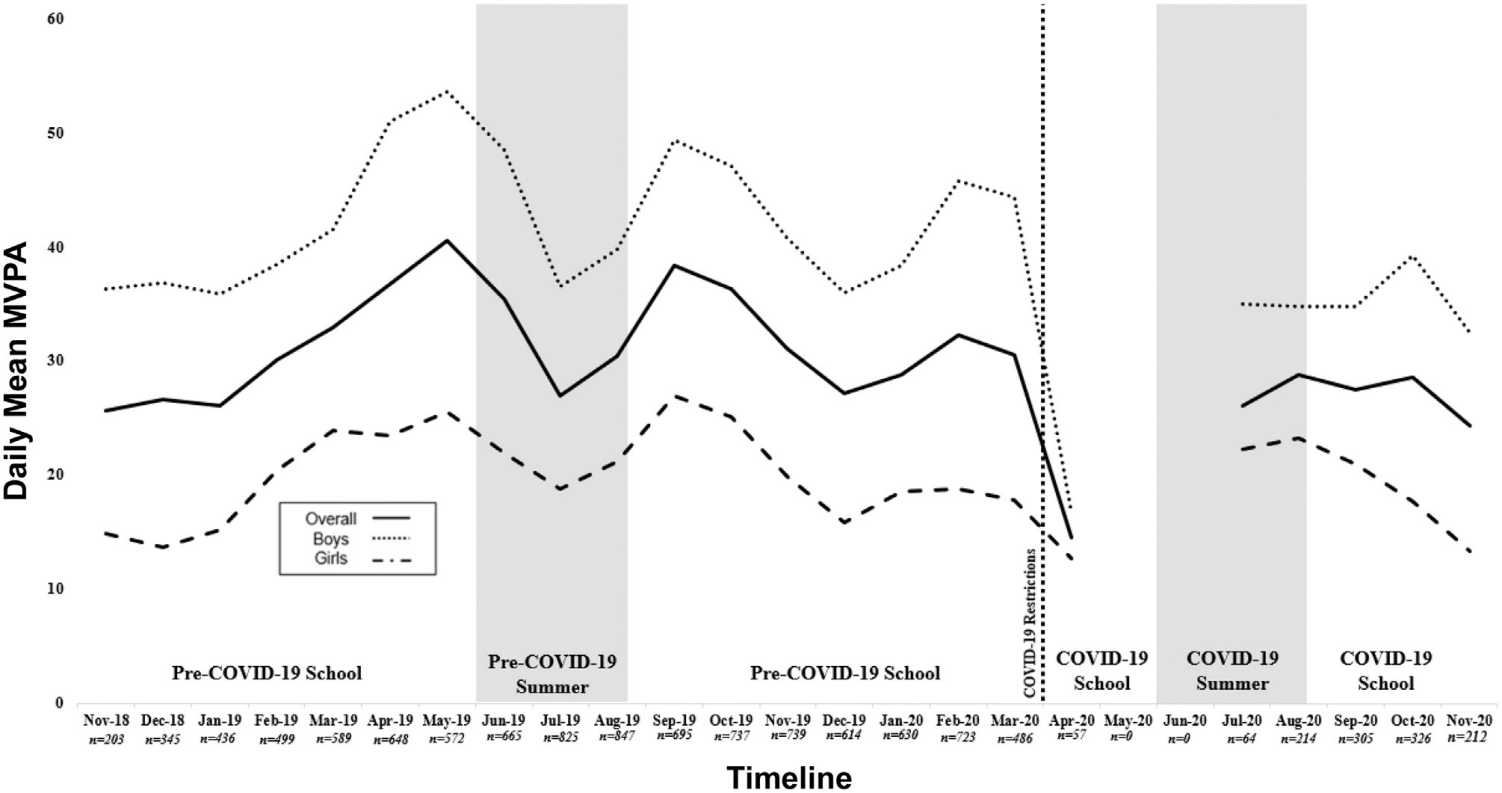
Description of conditions analyzed with COVID-19 restrictions depicted by daily mean MVPA. *Notes:* Figure is depicted by daily mean steps stratified by sex. Figure is depicted with monthly sample size. May 2020 and June 2020 recorded 0 participants. MVPA, moderate-to-vigorous physical activity.

This study utilized cross-sectional activity data from Year 2 (2018–2020) of the Adolescent Brain Cognitive Development (ABCD) study (4.0 release; 2018–2020), which involved participants aged 10–14 years. The data set is publicly available and deidentified. At the time of submission, the sponsor institution (UTHealth Austin) had been granted approval from the ABCD study (DAR ID: 12420) as well as the UTHealth IRB (IRB 232969). The ABCD study is the largest long-term investigation of brain development and health in the U.S. The study sample, recruitment process, and procedures have been previously described.^23^, ^24^, ^25^ Overall, a nationally distributed set of 21 sites were chosen through application for the study, and a sampling of schools within defined catchment areas for each site was conducted. Youth had to be aged 9 or 10 years at the time of their baseline assessment, all of which occurred between September 1, 2016 and August 31, 2018.^24^ Activity data analyzed were collected by Fitbit Charge 2 devices during Year 2 of the ABCD study between November 2018 and November 2020. Consumer-based devices such as the Fitbit Charge 2 have been found to be valid and reliable for assessing PA behavior among children and adolescents.^26^, ^27^, ^28^, ^29^ Throughout the 2-year interval of the Year 2 collection period, a rolling wear protocol was implemented where each site sequentially assigned to and retrieved devices from participants at different times. Of the original 11,500 participants recruited at baseline, approximately 7,000 were recruited to receive Fitbit devices to be worn for 21 days, which were distributed and worn. Figure 1 illustrates a timeline and monthly sample size of participants in the study who received devices illustrated by minutes per day of average MVPA. Additional figures illustrating daily mean steps and sedentary time can be found in Appendix Figures 1 and 2 (available online).

### Measures

In the U.S., state and local authorities (rather than federal) implemented restrictions and stay-at-home orders; therefore, no one date of restriction could be used to help extrapolate the period before/after COVID-19 shutdown or stay-at-home orders were implemented. To inform analyses, the authors compiled dates from outlets that created a timeline of school closures^30^ and thus placed COVID-19 restrictions between April 1, 2020 and November 27, 2020. The researchers also identified summer break and the school year for all collections between June 1–August 14 and August 15–May 31, respectively. Figure 1 also represents a visual representation of the timeline with COVID-19 restrictions and summer break months.

In terms of dependent variables, during 3 weeks (21 days) between November 2018 and November 2020, Fitbit Charge 2 devices (Fitbit Inc., San Francisco, CA) collected daily nonsleep steps, MVPA, and sedentary time data across sites. Previous research has demonstrated the reliability and accuracy of Fitbit devices in estimating accumulated PA.^26^^,^^31^ The authors also adopted the data extraction, filtering, and processing protocols established by the ABCD study, including PA thresholds.^31^ Thresholds for activity were classified as follows: sedentary defined as the number of minutes of sedentary (<1.5 METs) time observed in all valid minutes after exclusions, moderate activity defined as number of minutes moderately active (3–5.9 METs) time observed in all valid minutes after exclusions, and vigorous activity defined as number of minutes vigorously active (>6 METs) time observed in all valid minutes after exclusions. To compile MVPA, moderate and vigorous activity were combined into 1 variable. Following other studies, participants were included in the final analysis if their device registered days with >599 minutes of recorded wake wear time within each participant's 3-week study period, with a minimum requirement of 1,000 steps per day.^25^^,^^32^^,^^33^

In terms of independent variables, participants or their parent/caregivers self-reported their sex (female or male), race/ethnicity, household income, and age at baseline interviews. These characteristics have previously been identified as factors associated with levels of PA; therefore, they have been incorporated into this study as covariates.^34^

### Statistical Analysis

All statistical analyses were performed throughout 2022 and 2023 using Stata software, Version 17 (StataCorp, College Station, TX). For generalizability, propensity weights were applied to match key sociodemographic variables in the ABCD study to the American Community Survey from the U.S. Census.^25^ Mixed-effects models with random effects for participants were used to estimate associations of sociodemographic factors with repeated measures of daily steps, MVPA, and sedentary time analyzed as separate outcomes. The authors also conducted an exploratory analysis where they tested for effect modification by including an interaction term for sex and time, given evidence suggesting sex differences in maturation.^35^

## RESULTS

A total of 7,179 adolescents were included in the final analyses. Participants were included if they had all sociodemographic and covariate data and their devices registered days with >599 minutes of recorded wake wear time within each participant's 3-week study period, with a minimum requirement of 1,000 steps per day. Table 1 describes the sociodemographic variables included in the presented analyses. In total, individual sample sizes for each condition were as follows: *n*=5,569 for pre–COVID-19 school; *n*=1,419 for pre–COVID-19 summer; *n*=746 for COVID-19 school; and *n*=126 for COVID-19 summer. The analytic sample consisted of approximately 51% girls and 49% boys. Mean age of the total sample was 12.0 years (95% CI=11.9, 12.0). Approximately 50% of the final sample self-reported as a racial/ethnic minority (Black, Hispanic, multiracial, or other). Furthermore, self-reported combined household income was evenly distributed across the study population. Throughout the data collection window, adolescents accumulated 8,671.0 steps per day (95% CI=8,544.7; 8,797.3). After stratifying by sex, girls accumulated 7,949.7 steps (95% CI=7,841.4; 8,057.0), and boys accumulated 9,365.2 steps (95% CI=9,199.1; 9,531.2) daily. On average, the total sample accumulated 31.5 minutes of MVPA per day (95% CI=30.8, 32.3), and after stratifying by sex, girls accumulated 20.6 (95% CI=19.8, 21.3) minutes of MVPA per day, and boys accumulated 42.1 (95% CI=41.1, 43.0) minutes of MVPA per day. Finally, on average, the total sample was sedentary for 507.2 minutes (95% CI=504.2, 510.2) per day. After stratifying by sex, girls and boys were sedentary for 510.0 (95% CI=506.9, 513.0) and 504.6 (95% CI=499.9 509.3) minutes per day, respectively.

**Table 1:** Sociodemographic and Physical Activity Estimates of Study Participants (N= 7,179)

Variable	Mean	95% CI	
Sex (%)			
Girls (*n*=3,467)	51.0%	(49.2%,	52.7%)
Boys (*n*=3,712)	49.0%	(47.3%,	50.8%)
Mean age (years)			
Total	12.0	(11.9,	12.0)
Girls	11.9	(11.9,	12.0)
Boys	12.0	(12.0,	12.0)
Race/ethnicity (%)			
White	51.8%	(40.1%,	63.3%)
Black	15.5%	(10.5%,	22.4%)
Hispanic	21.0%	(11.2%,	35.7%)
Multiple	5.1%	(3.3%,	7.8%)
Other	3.2%	(1.5%,	6.8%)
Refused or unknown	3.4%	(2.4%,	4.8%)
Combined household income (%)			
<$25,000	17.6%	(13.8%,	22.3%)
$25,000–$50,000	18.8%	(15.6%,	22.4%)
$50,000–$75,000	15.5%	(13.9%,	17.1%)
$75,000–$99,000	12.4%	(10.5%,	14.4%)
$100,000–$199,000	20.3%	(16.3%,	25.2%)
≥$200,000	6.5%	(4.7%,	8.9%)
Refused or unknown	9.0%	(7.3%,	11.0%)
Mean steps (per day)			
Total	8,671.0	(8,544.7,	8,797.3)
Girls	7,949.7	(7,841.4,	8,058.0)
Boys	9,365.2	(9,199.1,	9,531.2)
Mean MVPA (minutes per day)			
Total	31.5	(30.8,	32.3)
Girls	20.6	(19.8,	21.3)
Boys	42.1	(41.1,	43.0)
Mean sedentary (minutes per day)			
Total	507.2	(504.2,	510.2)
Girls	510.0	(506.9,	513.0)
Boys	504.6	(499.9,	509.3)

*Note:* ABCD propensity weights were applied for all estimates on the basis of the American Community Survey from the U.S. Census.

ABCD, Adolescent Brain Cognitive Development; MVPA, moderate-to-vigorous physical activity.

Table 2 describes daily mean steps, MVPA, and sedentary time stratified by sex and condition, including mean wear days per individual by condition. Adolescents accumulated more mean daily steps during school months before COVID-19 lockdowns than during school months during COVID-19 lockdowns (8,999.7 vs 7,229.0, respectively). Adolescents also accumulated more mean daily minutes of MVPA during school months before COVID-19 lockdowns than during school months during COVID-19 lockdowns (32.4 vs 26.9, respectively). Adolescents accumulated fewer mean daily minutes of sedentary time during school months before COVID-19 lockdowns than during school months during COVID-19 lockdowns (505.9 vs 556.5, respectively). When comparing the summer months, adolescents accumulated more daily mean steps before COVID-19 than during COVID-19 (7,972.2 vs 7,511.0, respectively). Descriptively, adolescents accumulated amounts of minutes of MVPA during summer months before COVID-19 similar to that during COVID-19 (29.9 vs 29.2, respectively). Adolescents appear to have accumulated less sedentary time in minutes during summer months before COVID-19 than during COVID-19 (486.3 vs 500.9).

**Table 2:** Mean Estimates of Steps, MVPA, and Sedentary Time of Analyzed Sample

Outcome			Total					Girls					Boys		
	*n*	Wear days	Mean	95% CI		*n*	Wear days	Mean	95% CI		*n*	Wear days	Mean	95% CI	
Steps (per day)															
Pre–COVID-19 school	5,569	16.8	8,999.7	(8,880.3;	9,119.1)	2,648	17.1	8,257.3	(8,161.1;	8,353.6)	2,921	16.5	9,692.4	(9,519.9;	9,864.9)
Pre–COVID-19 summer	1,419	13.9	7,972.2	(7,719.7;	8,224.8)	724	14.1	7,340.4	(7,018.7;	7,662.0)	695	13.7	8,649.6	(8,336.6;	8,962.6)
COVID-19 school	746	14.5	7,229.0	(6,859.2;	7,598.9)	355	14.5	6,497.9	(6,139.1;	6,856.6)	391	14.5	7,929.8	(7,441.4;	8,418.3)
COVID-19 summer	126	11.0	7,511.0	(6,906.7;	8,115.2	71	12.7	7,846.4	(7,075.2;	8,617.6)	55	8.8	6,915.0	(5,293.5;	8,536.4)
MVPA (minutes/day)															
Pre–COVID-19 school			32.4	(31.5,	33.3)			20.9	(20.2,	21.6)			43.2	(42.0,	44.3)
Pre–COVID-19 summer			29.9	(28.3,	31.5)			19.9	(18.5,	21.3)			40.6	(38.4,	42.9)
COVID-19 school			26.9	(24.3,	29.4)			18.0	(16.3,	19.8)			35.3	(31.5,	39.2)
COVID-19 summer			29.2	(24.8,	33.7)			26.3	(20.6,	31.9)			34.5	(26.1,	43.0)
Sedentary (minutes/day)															
Pre–COVID-19 school			505.9	(502.6,	509.2)			509.6	(506.2,	512.9)			502.5	(497.7,	507.3)
Pre–COVID-19 summer			486.3	(478.4,	494.2)			487.9	(478.6,	497.2)			484.6	(472.9,	496.2)
COVID-19 school			556.5	(548.4,	564.7)			558.7	(546.9,	570.5)			554.4	(542.7,	566.2)
COVID-19 summer			500.9	(472.1,	529.7)			486.7	(457.5,	516.0)			526.0	(462.8,	589.2)

*Note:* Wear days are expressed as mean days of valid wear per individual per condition.

Dates for conditions are as follows:

Pre–COVID-19 school: October 20, 2018–May 31, 2019 and August 15, 2019–March 31, 2020.

Pre–COVID-19 summer: June 1, 2019–August 14, 2019.

COVID-19 school: April 1, 2020–May 31, 2020 and August 15, 2020–November 27, 2020.

COVID-19 summer: June 1, 2020–August 14, 2020.

*n*s for each condition are the same for all behaviors as they are not listed. ABCD propensity weights were applied for all estimates on the basis of the American Community Survey from the U.S. Census.

ABCD, Adolescent Brain Cognitive Development; MVPA, moderate-to-vigorous physical activity.

Table 3 describes fully adjusted regression models by behavior with additional linear combinations comparisons. After controlling for all covariates, before COVID-19 lockdowns, adolescents accumulated significantly fewer steps during summer months than during school months (coefficient= −705.6 steps; 95% CI= −879.6, −531.6). During school months, adolescents also accumulated significantly fewer steps during COVID-19 lockdowns than before COVID-19 lockdowns (coefficient= −1,782.3 steps; 95% CI= −2,052.7; −1,511.8). Before COVID-19 lockdowns, the overall sample also accumulated significantly fewer steps during summer than during school months (coefficient= −1,960.7 steps; 95% CI= −2,444.7; −1,476.7). When examining the linear combinations in the model, during the summer months, adolescents accumulated significantly more mean daily steps than during COVID-19 lockdowns (coefficient=1,255.1 steps; 95% CI=745.3; 1,765.0). Table 3 also highlights how a similar trend was found when stratifying models by sex.

**Table 3:** Mixed-Effects Linear Regressions Examining the Association Between Steps, MVPA, and Sedentary Time by Condition, Fully Adjusted

Outcome	Overall				Girls				Boys			
	Coefficient	*p*-value	(95% CI)		Coefficient	*p*-value	(95% CI)		Coefficient	*p*-value	(95% CI)	
Steps (per day)												
Pre–COVID-19 summer	**−705.6**	**<0.01**	**(−879.6,**	**−531.6)**	**−590.7**	**<0.01**	**(−837.6,**	**−343.7)**	**−844.2**	**<0.01**	**(−1,081.2;**	**−607.2)**
COVID-19 school	**−1,782.3**	**<0.01**	**(−2,052.7;**	**−1,511.8)**	**−1,669.7**	**<0.01**	**(−2,019.9;**	**−1,319.5)**	**−1,942.0**	**<0.01**	**(−2,349.4;**	**−1,534.7)**
COVID-19 summer	**−1,960.7**	**<0.01**	**(−2,444.7;**	**−1,476.7)**	**−1,216.8**	**<0.01**	**(−1,843.4;**	**−590.3)**	**−2,855.7**	**<0.01**	**(−3,562.3;**	**−2,149.0)**
Constant/reference (Pre–COVID-19 school)	10,118.6	0.00	(8,927.4;	11,309.8)	9,244.2	0.00	(7,755.8;	10,732.5)	9,802.2	0.00	(7,988.2;	11,616.3)
*LINCOM*												
Pre–COVID-19 Summer versus COVID-19 summer (ref)	**1,255.1**	**<0.01**	**(745.3;**	**1,765.0)**	626.2	0.07	(**−**40.6;	1,293.0)	**2,011.5**	**<0.01**	**(1,271.9;**	**2,751.0)**
COVID-19 school versus COVID-19 summer (ref)	178.5	0.43	(**−**268.2,	625.2)	**−**452.8	0.14	(**−**1,048.3;	142.7)	**913.6**	**<0.01**	**(267.6;**	**1,559.6)**
MVPA (minutes per day)												
Pre–COVID-19 Summer	**−1.5**	**0.02**	**(−2.6,**	**−0.3)**	**−**0.8	0.24	(**−**2.1,	0.5)	**−2.2**	**0.03**	**(−4.2,**	**−0.2)**
COVID-19 school	**−6.2**	**<0.01**	**(−8.4,**	**−4.0)**	**−2.6**	**0.02**	**(−4.8,**	**−0.4)**	**−10.0**	**<0.01**	**(−13.6,**	**−6.4)**
COVID-19 summer	**−**3.2	0.11	(**−**6.9,	0.6)	2.9	0.20	(**−**1.5,	7.3)	**−10.1**	**<0.01**	**(−16.4,**	**−3.9)**
Constant/reference (Pre–COVID-19 school)	16.7	0.00	(6.9,	26.5)	**−**5.4	0.30	(**−**15.6,	4.9)	17.5	0.00	(1.4,	33.5)
LINCOM												
Pre–COVID-19 summer versus COVID-19 summer (ref)	1.7	0.40	(**−**2.2,	5.6)	**−**3.6	0.12	(**−**8.2,	0.9)	**7.9**	**0.02**	**(1.4,**	**14.4)**
COVID-19 school versus COVID-19 summer (ref)	**−**3.1	0.09	(**−**6.6,	0.5)	**−5.5**	**0.01**	**(−9.8,**	**−1.1)**	0.1	0.97	(**−**5.6,	5.8)
Sedentary (minutes per day)												
Pre–COVID-19 summer	**−16.8**	**<0.01**	**(−22.0,**	**−11.6)**	**−20.7**	**<0.01**	**(−27.7,**	**−13.6)**	**−12.7**	**<0.01**	**(−20.3,**	**−5.0)**
COVID-19 school	**29.6**	**<0.01**	**(18.9,**	**40.3)**	**31.3**	**<0.01**	**(16.4,**	**46.1)**	**28.1**	**<0.01**	**(12.8,**	**43.4)**
COVID-19 summer	**−20.3**	**0.03**	**(−38.5,**	**−2.1)**	**−**16.2	0.15	(**−**38.0,	5.6)	**−**23.1	0.14	(**−**53.6,	7.4)
Constant/reference (Pre–COVID-19 school)	275.1	0.00	(233.1,	317.1)	249.2	0.00	(189.8,	308.6)	293.5	0.00	(233.7,	353.4)
LINCOM												
Pre–COVID-19 summer versus COVID-19 summer (ref)	2.3	0.81	(**−**16.4,	21.0)	**−**4.5	0.70	(**−**27.2,	18.3)	10.4	0.51	(**−**20.8,	41.7)
COVID-19 school versus COVID-19 summer (ref)	**48.6**	**<0.01**	**(31.8,**	**65.3)**	**47.4**	**<0.01**	**(27.5,**	**67.3)**	**51.2**	**<0.01**	**(22.8,**	**79.7)**

*Note:* Boldface indicates statistical significance (*p*<0.05).

LINCOM refers to additional estimates of parameters that are not directly provided in the base regression output. ABCD propensity weights are applied for all estimates on the basis of the American Community Survey from the U.S. Census. All models utilized GEE, which explicitly models the clustering of observations within individuals. Model-derived estimates included the following variables as covariates: weekend, time (monthly), race/ethnicity, household income, child age, and study site. Overall model for each behavior also includes a sex-by-month interaction term.

ABCD, Adolescent Brain Cognitive Development; GEE, generalized estimating equation; LINCOM, Linear Combination; MVPA, moderate-to-vigorous physical activity.

Before COVID-19 lockdowns, adolescents accumulated significantly fewer minutes of MVPA during summer months than during school months (coefficient= −1.5 minutes; 95% CI= −2.6, −0.3). During school months, adolescents also accumulated significantly fewer minutes of MVPA during COVID-19 lockdowns than before COVID-19 lockdowns (coefficient= −6.2 minutes; 95% CI= −8.4, −4.0). Unlike steps, there were no model-derived differences in minutes of MVPA when examining COVID-19 summer versus pre–COVID-19 school. When stratifying models by sex, during school months, girls and boys accumulated significantly fewer minutes of MVPA during COVID-19 lockdowns than before COVID-19 lockdowns (coefficient= −2.6 steps; 95% CI= −4.8, −0.4 and coefficient= −2.2 minutes; 95% CI= −4.2, −0.2, respectively). During school months, boys also accumulated significantly fewer minutes of MVPA during COVID-19 lockdowns than before COVID-19 lockdowns (coefficient= −10.0 minutes; 95% CI= −13.6, −6.4). Boys also accumulated significantly fewer minutes of MVPA during summer months during COVID-19 lockdowns than during school months before COVID-19 lockdowns (coefficient= −10.1 minutes; 95% CI= −16.4, −3.9). When examining the linear combinations in the model, during COVID-19, girls accumulated significantly fewer minutes of MVPA during school months than during summer months (coefficient= −5.5 minutes; 95% CI= −9.8, −1.1). Furthermore, during summer months, boys accumulated significantly more minutes of MVPA before COVID-19 lockdowns (coefficient=7.9 minutes; 95% CI=1.4, 14.4).

Before COVID-19 lockdowns, adolescents accumulated significantly fewer minutes of sedentary time during summer months than during school months (coefficient= −16.8 minutes; 95% CI= −22.0, −11.6). During school months, adolescents accumulated significantly more minutes of sedentary time during COVID-19 lockdowns than before COVID-19 lockdowns (coefficient=29.6 minutes; 95% CI=18.9, 40.3). Before COVID-19 lockdowns, the overall sample also accumulated significantly fewer minutes of sedentary time during summer than during school months (coefficient= −20.3 minutes; 95% CI= −38.5, −2.1). When stratifying models by sex, before COVID-19 lockdowns, both girls and boys accumulated significantly fewer minutes of sedentary time during summer months than during school months (coefficient= −20.7 minutes; 95% CI= −27.7, −13.6 and coefficient= −12.7 minutes; 95% CI= −20.3, −5.0, respectively). During school months, girls and boys accumulated significantly more minutes of sedentary time during COVID-19 lockdowns than before COVID-19 lockdowns (coefficient=31.3 minutes; 95% CI=16.4, 46.1 and coefficient=28.1 minutes; 95% CI=12.8, 43.4, respectively). When examining the linear combinations in the model, during COVID-19, adolescents accumulated significantly more minutes of sedentary time during school months than during summer months (coefficient=48.6 minutes; 95% CI=31.8, 65.3). Findings were similar by sex as well. Across all models, no significant interactions were observed when examining sex-by-month interaction term.

## DISCUSSION

This study aimed to examine the patterns of PA among a population-based sample of U.S. adolescents over a 2-year period spanning before and during the COVID-19 pandemic. Furthermore, the authors aimed to extrapolate the potential double jeopardy of COVID-19 and summer on objectively measured PA. When examining overall activity before and during the pandemic, findings align with and support other work concluding a decrease in overall activity from children and adolescents.^36^, ^37^, ^38^ There was also a significant decrease in mean daily steps and MVPA as well as a significant increase in sedentary time during school months when COVID-19 restrictions were in place between April and November 2020. When stratifying by sex, both boys and girls experienced significant decreases in their daily steps and daily minutes of MVPA during school months when COVID-19 restrictions were in place compared with that before the pandemic. These findings add to the body of evidence and reiterate the impact that closing schools and school-like programs for prolonged periods of time can have on the PA levels of adolescents in the U.S.

This study's unique approach was exploring the potential compounding effect of summer vacation and the COVID-19 pandemic on PA levels. It was hypothesized that summer months during COVID-19 restrictions would be the period when adolescents in the U.S. would be the least active. This was based on the premise of the structured days hypothesis (SDH), which posits that access to structured programs over summer is lower than the consistency and availability of school and school-based programs during the 9-month school year, and this can lead to lower levels of PA in youth. The SDH posits that school days are fundamentally different from less-structured days, such as a weekend day or summer days because they consistently contain a daily structure and routine with intentional (e.g., recess, physical education, before/after school programs, organized sports programs) and unintentional (e.g., regular transitions between activities, walking to school) PA opportunities provided to most children throughout the day. Summer does not necessarily contain this same dose of consistency or exposure, and the addition of COVID-19 restrictions to this already less-structured time was hypothesized to cause a double jeopardy effect on adolescents PA. Authors’ data show that adolescents accumulated over 1,200 more daily steps during the summer months before COVID-19 than during summer months during COVID-19. This finding was most likely due to the differences in activity by sex because boys accumulated over 2,000 more daily steps during the summer months before COVID-19 than during the summer months during COVID-19. Furthermore, boys accumulated nearly 8 more minutes of daily MVPA during summer months before COVID-19 than during summer months during COVID-19. These findings are alarming because they confirm a double jeopardy effect with summer and COVID-19. Authors’ data highlight the protective effect on adolescents’ PA that structured settings can offer in a consistent and routine nature.^3^ Evidence surrounding the SDH consistently indicates that adolescents accumulate less PA during days with less structure.^6^^,^^39^^,^^40^ These significant differences in activity, which were more pronounced among boys, not only confirm how the presence of structured settings is correlated with behaviors but also confirm how restrictions due to COVID-19 impacted the behaviors of boys during a critically important developmental stage.

Other findings surrounding overall activity were mixed and did not align with our original hypothesis. No differences in sedentary time were found when comparing summers before and during COVID-19. Furthermore, across the study sample, during COVID-19, individuals were more sedentary during school months than during summer months. One potential reason for the increased sedentary time during COVID-19 school months may be due to classes being offered online during the early stages of COVID-19, which would limit overall activity and increase sedentary time. Once classes resumed, social distancing within the schools may have also limited activity, potentially increasing sedentary time.^21^

The present study's findings may help explain findings related to health outcomes examined during COVID-19. Several studies have concluded significant increases in the prevalence of obesity, BMI, or zBMI throughout the COVID-19 pandemic.^41^, ^42^, ^43^, ^44^, ^45^, ^46^ Notably, a recent systematic review and meta-analysis concluded that although there were clinically significant increases in weight gain, BMI, and obesity among children and adults, increases were greater in children.^42^ Reasons why obesity-contributing behaviors worsened throughout the COVID-19 pandemic are likely attributable to a complex interplay of variables such as SES and access to healthy-structured programming. Still, the cessation of school-based activities to mitigate viral spread is likely a significant contributing factor.^47^ Concepts of SDH, such as incorporating preplanned, compulsory, adult-supervised activities into a young person's day, positively impact these behaviors.^3^^,^^4^^,^^7^^,^^8^^,^^48^^,^^49^ Many aspects of the SDH were removed during the pandemic, inevitably impacting students’ ability to engage in behaviors that positively shape health outcomes, thus potentially illustrating how COVID-19 was a large natural experiment of the SDH.^14^

This study has several strengths. First, the large population-based sample size recruiting children from across the U.S. allows for strong inference when examining the impacts of COVID-19 on PA. Second, using objectively measured data in which participants wore devices for 21 days is unique because many studies examining outcomes related to COVID-19 have relied on self-report questionnaires to measure behaviors, which can introduce measurement errors due to social desirability bias and recall errors.^50^

### Limitations

Results should also be interpreted in the context of the study's limitations. The cross-sectional design of the study limits the ability to draw causal inferences. Furthermore, the sample sizes in each examined condition were not equally distributed. The study design and distribution of devices were not explicitly structured to capture these conditions, and each site faced different challenges in Fitbit distribution during the COVID-19 lockdowns; therefore, individual characteristics may have been different pre–COVID-19 from the current analysis. Thus, the unequal distribution of individuals in each condition is a limitation that should be accounted for when interpreting findings. However, in the presented analysis, the smallest condition (COVID-19 summer) produced approximately 1,400 individual observation days with valid PA data. Follow-up studies are needed to understand whether adolescents experienced a rebound in their engagement of obesity-contributing behaviors after the lift of COVID-19 restrictions that also incorporates study design features to capture and examine periods with less structure (i.e., summer breaks).^14^

## CONCLUSIONS

The findings from this study indicate how the COVID-19 pandemic may have negatively impacted the activity behaviors of U.S. children during early adolescence. These data could inform future interventions to promote health behaviors and specifically provide guidance on when and where resources could be allocated throughout a calendar year as it relates to the importance of structured PA opportunities. Finally, this study highlighted the impact of summer break on PA and sedentary time during and before the pandemic.

## Appendix Supplementary materials

Image, application 1

## Footnotes

Supplementary material associated with this article can be found in the online version at doi:10.1016/j.focus.2024.100253.
