# Supplementary material for: Physical Activity and Sedentary Time Among U.S. Adolescents Before and During COVID-19: Findings From a Large Cohort Study
Source: AJPM Focus. 2024 Jun 17;3(5):100253. doi: 10.1016/j.focus.2024.100253 (PMC11340494; doi:10.1016/j.focus.2024.100253)

Appendix Material

Physical Activity and Sedentary Time Among U.S. Adolescents Before and During COVID-19: Findings from a Large Cohort Study

AJPM Focus

**Appendix Figure 1.** Description of conditions analyzed with COVID-19 restrictions depicted by mean steps


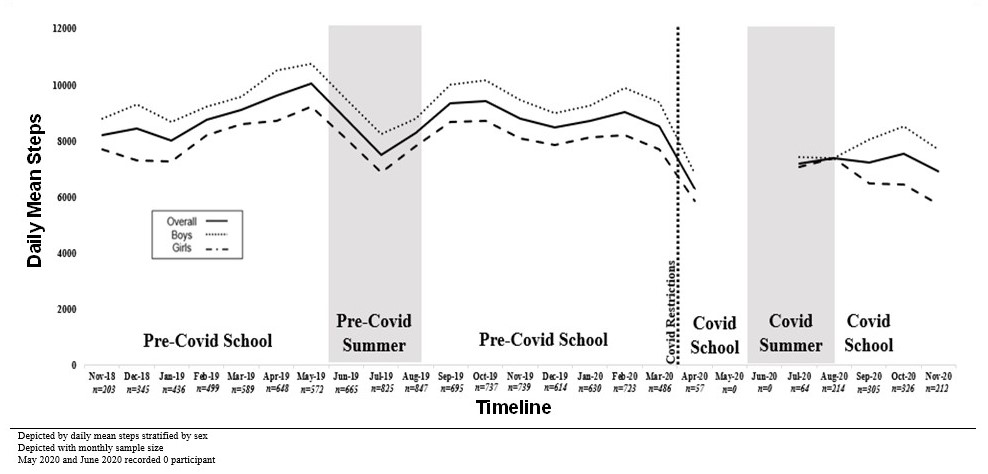


**Appendix Figure 2.** Description of conditions analyzed with COVID-19 restrictions depicted by mean sedentary time


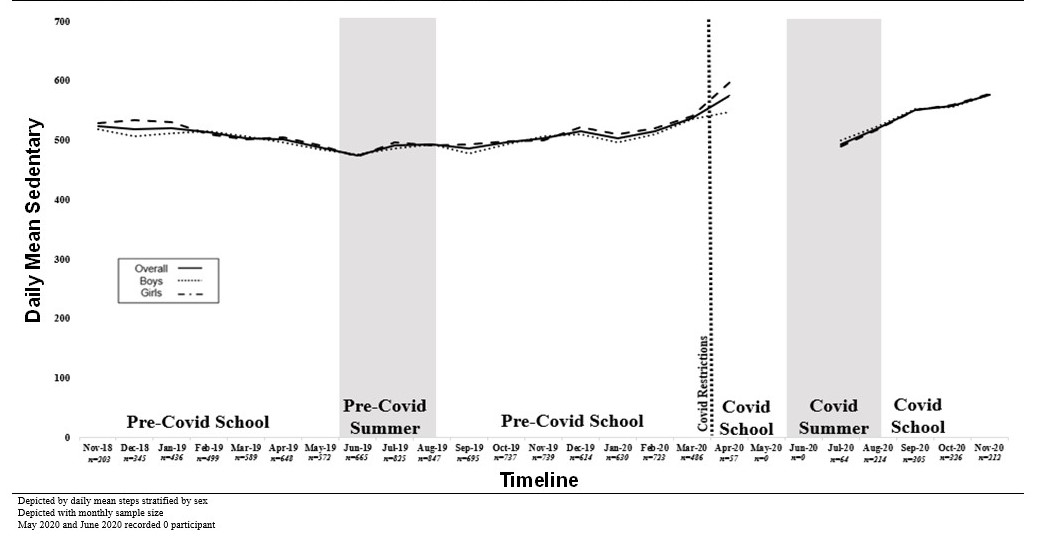

Supplement: Supplementary file 1 [file mmc1.docx]
